# Bayesian Multilevel Analysis of Utilization of Antenatal Care Services in Ethiopia

**DOI:** 10.1155/2020/8749753

**Published:** 2020-07-04

**Authors:** Cheru Atsmegiorgis Kitabo, Ehit Tesfu Damtie

**Affiliations:** ^1^Department of Statistics, College of Natural and Computational Sciences, Hawassa University, Hawassa, Ethiopia; ^2^Department of Statistics, College of Natural and Computational Sciences, Dilla University, Dilla, Ethiopia

## Abstract

In sub-Saharan Africa, 72% of pregnant women received an antenatal care visit at least once in their pregnancy period. Ethiopia has one of the highest rates of maternal mortality in sub-Saharan African countries. So, this high maternal mortality levels remain a major public health problem. According to EDHS, 2016, the antenatal care (ANC), delivery care (DC), and postnatal care (PNC) were 62%, 73%, and 13%, respectively, indicating that ANC is in a low level. The main objective of this study was to examine the factors that affect the utilization of antenatal care services in Ethiopia using Bayesian multilevel logistic regression models. The data used for this study comes from the 2016 Ethiopian Demographic and Health Survey which was conducted by the Central Statistical Agency (CSA). The statistical method of data analysis used for this study is the Bayesian multilevel binary logistic regression model in general and the Bayesian multilevel logistic regression for the random coefficient model in particular. The convergences of parameters are estimated by using Markov chain Monte-Carlo (MCMC) using SPSS and MLwiN software. The descriptive result revealed that out of the 7171 women who are supposed to use ANC services, 2479 (34.6%) women were not receiving ANC services, while 4692 (65.4%) women were receiving ANC services. Moreover, women in the Somali and Afar regions are the least users of ANC. Using the Bayesian multilevel binary logistic regression of random coefficient model factors, place of residence, religion, educational attainment of women, husband educational level, employment status of husband, beat, household wealth index, and birth order were found to be the significant factors for usage of ANC. Regional variation in the usage of ANC was significant.

## 1. Background of the Study

Maternal health refers to the well-being of women through pregnancy, childbirth, and the postpartum period. In rich countries, where women have access to basic health care, giving birth is a positive and fulfilling experience. On the other hand, for many women that live in poor countries, it is associated with suffering, ill-health, and even death [1]. Maternal health is advantageous to the communities, families, and nation due to its profound effects on the health of women, immediate contact of survival of the newborn, and long-term well-being of children, particularly girls and the well-being of families.

The recent World Health Organization (WHO) estimates on maternal mortality showed that developed countries had a consistent low maternal mortality ratio that averaged less than 10 deaths per 100,000 live births for over a decade [2]. Studies showed that Poland, Sweden, Japan, United Kingdom, and New Zealand have had the best performance in reducing and maintaining low maternal mortality levels over the past decades [3].

In sub-Saharan Africa, there are still more maternal health care problems. It is approximated that about 800 girls and women died every day as a result of pregnancy and childbirth-related complications [4]. Maternal deaths are now increasingly concentrated in sub-Saharan Africa, where high fertility rates combine with inadequate access to quality antenatal care and skilled attendance at birth to substantially elevate the risk of death in this region. The WHO estimates that over 500,000 women and girls die from complications of pregnancy and childbirth each year, worldwide, with approximately 99% of these deaths occurring in developing countries [5].

In sub-Saharan Africa, countries like Burundi, Cameroon, and Senegal registered about 740, 590, and 320 deaths per 100,000 live births, respectively. Variations in maternal health outcomes and any issues in sub-Saharan Africa have attributed to pregnancy and childbirth complications with unbalanced socioeconomic status [6].

The use of antenatal care services among women in sub-Saharan Africa has shown that 72% of pregnant women received an antenatal care visit once or more times. The very low maternal and infant morbidity and mortality rates reported for developed countries compared with the high figures in developing countries have been attributed to the higher utilization of modern maternal health services by the former [7].

Ethiopia has one of the highest rates of maternal mortality in sub-Saharan African countries. So, these high maternal mortality levels remain a major public health problem. The proportion of women reproductive age from 15 to 49 reports having problems in accessing health care services, i.e, the services are decreased from 96% in 2005, 94% in 2011, and 70% in 2016, despite the country's quest to reduce maternal mortality of ratio to 199 per 100,000 live births by 2020 based on the suggestion made with the goal of the reproductive health program. According to the Ethiopian Demographic and Health Survey, the antenatal care (ANC), skilled delivery care, and postnatal care (PNC) were 62%, 73%, and 13%, respectively, indicating that ANC is low [8].

Considering the goal of reducing maternal mortality and improving maternal health care services, it is essential to understand the factors that affect antenatal care service utilization in order to find the possible solutions for improving maternal health care service in Ethiopia.

Studies conducted in Nigeria and Ethiopia tried their effort to establish correlates having an effect on antenatal care service utilization using binary logistic regression [9]. A study conducted by [9] which used logistic regression to study the utilization of maternal health care services in Kenya found out a number of socioeconomic and demographic characteristics of women greatly influence the usage of maternal health care services such as age of women, education, work status, place of residence, birth order, wealth index, and region. However, their study did not investigate the relative contribution at the individual and regional level of factors influencing women's use of health care services using the Bayesian multilevel.

Despite the country's mission to reduce maternal mortality ratio to 199 maternal deaths per 100,000 live births by 2020 on the basis of the recommendation made with the goal of the reproductive health program, the ratio is still very high [8]. This gap needs to be investigated to ensure an increase in the utilization of maternal health care among mothers through identifying independent variables influencing the utilization of antenatal care services, hence a decrease in child mortality and improvement in maternal care. To fill this gap, the Bayesian multilevel modeling has been employed for identifying factors affecting antenatal care service utilization in Ethiopia at individual and regional levels.

There are a number of reasons for using multilevel models. The classical logistic regression analysis treats the units of analysis as independent observations. One consequence of not considering hierarchical structures is that standard errors of regression coefficients will be underestimated, leading to an overstatement of statistical significance and wrong conclusion. Standard errors for the coefficients of higher-level predictor variables will be the most affected by ignoring grouping.

In a situation where research questions concern the extent of grouping in individual outcomes, and the identification of “outlying” groups, multilevel analysis is highly advised. For evaluating health care intervention, obtaining the effect of antenatal care on maternal mortality can be examined. Additionally, for estimating group effects simultaneously with the effects of group-level predictors, the use of multilevel analysis is mandatory. Moreover, making an inference to a population of groups can be attained using the multilevel analysis [10]. The multilevel analysis enables the proper investigation of the effects of all explanatory variables measured at different levels on the response variable. They employed the model on HIV testing and got a more accurate result [11].

The 2016 Ethiopian Demographic and Health Survey (EDHS) sample was a stratified cluster and selected in two stages [8]. This stratified cluster sampling scheme often introduces multilevel dependency or correlation among the observations that can have implications for model parameter estimates. For multistage clustered samples, the dependence among observations often comes from several levels of the hierarchy. In this case, the use of single-level statistical models is no longer valid and reasonable. Hence, in order to draw appropriate inferences and conclusions from multistage stratified clustered survey data, we may require techniques like multilevel modeling [12].

The purpose of this study is to understand the current status of utilization of antenatal care services in Ethiopia and to elucidate the various explanatory variables having an effect on the use of ANC services in Ethiopia. Moreover, the study is aimed at comparing the predictive performance of the Bayesian multilevel and the classical binary logistic regression models. The results of the study would help the policymakers in understanding the determinants of maternal and child mortality in the country and serve as an important input for any possible intervention aimed at improving the low utilization of antenatal care services in the country. This study covered women in the reproductive age from 15 to 49 years who gave the outcome of antenatal care service in Ethiopia.

## 2. Methodology

### 2.1. Study Area and Variable Description

Ethiopia has 9 regional states and two city administrations with 611 districts and 15,000 lower administrations. According to the 2016 Ethiopia Demographic and Health Survey (EDHS), the total population of Ethiopia was around 102 million with 4.5 fertility rates. Secondary data was extracted from the 2016 Ethiopian Demographic and Health Survey (EDHS). It is the fourth Demographic and Health Survey conducted from January 18, 2016, to June 27, 2016, by the Central Statistical Agency (CSA). From a total of 18,008 households, 16,650 were successfully interviewed. In the interviewed households, 16,583 eligible women were identified for individual interviews [8]. Interviews were completed for 15,683 women, and all women who are in the reproductive age group (aged from 15 to 49 years) were included.

#### 2.1.1. Correlates of Antenatal Care

On the basis of reviewed literatures, potential determinant factors that are expected to be correlated with antenatal care service utilization have been included. Variables considered in this study are categorized into dependent and independent variables.

*(1) Dependent Variable*. The outcome of interest in this study was the utilization of ANC service. A woman is considered to have used ANC if she was checked by a health professional at least once during her pregnancy period. Therefore, the outcome is a categorical variable which is measured as use or not use of service care.

(1) $$\begin{array}{c} Y_{ij}=\left\{\begin{array}{c} 1,\text{if the }i^{\text{th}}\text{ women use of service care in the }j^{\text{th}}\text{ region},\\ 0,\text{if the }i^{\text{th}}\text{ women not use of service care in the }j^{\text{th}}\text{ region}. \end{array}\right. \end{array}$$

With *P*_*ij*_ = *P* (*Y*_*ij*_ = 1/*X*_*ij*_) is the probability of using service care for woman *i* in region *j*, *X*_*ij*_ is the observed characteristic of the *i*^th^ women in the *j*^th^ region and 1 − *P*_*ij*_ = *P* (*Y*_*ij*_ = 0/*X*_*ij*_) is the probability of not using service care for woman *i* in region *j*.

*(2) Independent Variable*. The utilization of antenatal care services is influenced by socioeconomic and demographic factors. These include age of respondent employment status of women, employment status of the husband, birth order, husband's education status, place of residence, religion, attitudes towards wife-beating, women's decision-making power, marital status, mothers' educational status, women exposure to media, and wealth index.

### 2.2. Method of Statistical Data Analysis

#### 2.2.1. Multilevel Linear Models

When the data was collected in hierarchical or clustered structures, the suitable model is multilevel models. Multilevel models are used to account for the correlation of observations within a given group by incorporating group-specific random effects. These random effects can be nested (individuals nested in regions, with random effects at the women and region levels) [13]. The dependent variable must be examined at the lowest and highest level of analysis.

#### 2.2.2. Bayesian Multilevel Logistic Regression Model

*(1) Two-Level Model*. A multilevel logistic regression model can account for lack of independence across levels of nested data (i.e., individuals nested within regions). It assumes that all experimental units are independent meaning that any variable which affects the utilization of antenatal care services has the same effect in all regions, but multilevel models are used to assess whether the effect of predictors varies from region to region. The probability of “success” or “failure” is the same for all individuals in the group.

The standard assumption is that *Y*_*ij*_ has a Bernoulli distribution. Like the logistic regression, the *p*_*ij*_  is modeled using the link function logit. The two-level models are given by

(2) $$\begin{array}{c} \text{logit }\left(\;p_{ij}\right)=\log\;\left(\frac{p_{ij}}{1-p_{ij}}\right)=\beta_{o}+\overset{k}{\underset{h=1}{\sum }}\beta_{hj}x_{hij}+U_{0j}+\overset{k}{\underset{h=1}{\sum }}U_{hj}x_{hij}, \end{array}$$

where *X*_*hij*_ = (*X*_1*ij*_, *X*_2*ij*_, ⋯⋯., *X*_*kij*_) represents the first and the second level of covariates for variable *k*, *β* = (*β*_0_, *β*_1_, ⋯⋯⋯⋯., *β*_*k*_) are regression parameter coefficients, and *U*_0*j*_, *U*_1*j*_, ⋯⋯⋯⋯*U*_*kj*_ are the random cluster effects of a model parameter at higher levels with the assumption that follows a normal distribution with mean zero and variance *σ*_*u*_^2^. Therefore, conditional on *U*_0*j*_, *U*_1*j*_, ⋯⋯⋯*U*_*kj*_, the *Y*_*ij*_ can be assumed to be independently distributed as Bernoulli random variables.

*(i) Bayesian Multilevel Analysis of Empty Models*. The empty two-level model also called a null two-level model for a dichotomous outcome variable refers to a population of groups (level-2units) and specifies the probability distribution for group dependent probabilities *p*_*j*_ in *Y*_*ij*_=*p*_*j*_+*ε*_*ij*_ without taking further explanatory variables into account. We focus on the model that specifies the transformed probabilities *f*(*p*_*j*_) and the logit function; then, *f*(*p*_*j*_) is just the log-odds for group *j*. Thus, the log-odds have a normal distribution in the population of groups, and it is given by

(3) $$\begin{array}{c} f\;\left(p_{j}\right)=\beta_{0}+u_{0j}, \end{array}$$

where *p*_*j*_ = *e*^*β*_0_+*u*_0*j*_^/(1 + *e*^*β*_0_+*u*_0*j*_^), *β*_0_ is the population average of the transformed probabilities, and *u*_0*j*_ are regional level random effects that are i.i.d. normally distributed with zero means and constant variances  *σ*_*u*_^2^. This model does not include a separate parameter for the level-one variance, because the level-one residual variance of the *Y*_*ij*_  follows the Bernoulli distribution directly from the success probability, as indicated by Var(*ϵ*_*ij*_) = *p*_*j*_(1 − *p*_*j*_).

The likelihood function of the empty model is

(4) $$\begin{array}{c} L\;\left(\frac{p_{j}}{y_{ij}}\right)=\underset{j}{\prod }{\left(p_{ij}\right)}^{yij}{\left(1-p_{j}\right)}^{1-yij}. \end{array}$$

*(a) Prior Distribution*. For the parameters *β*_0_ and *σ*_*u*0_^2^, the prior distributions are default noninformative uniform distribution for the intercept and gamma for the random part of the model. It is given by *P* (*β*_0_) ∝ 1 and *P* (*σ*_*u*0_^−2^) ∝ gamma (*α*, *β*) where *α* and *β* are scale and shape parameters which are fixed constants.

*(b) Posterior Distribution*. By using MCMC methods, we can estimate random parameters from the posterior distribution *P* (*σ*_*u*0_^2^, *β*_0_/*y*_*ij*_).

For parameter *β*_0_, the full conditional distribution is

(5) $$\begin{array}{c} P\;\left(\frac{\beta_{0}}{\sigma_{u0}^{2}},y_{ij}\right)\propto \underset{ij}{\prod }{\left(\frac{e^{\beta_{0}+u_{0j}}}{1+e^{\beta_{0}+u_{0j}}}\right)}^{yij}{\left(\frac{1}{1+e^{\beta_{0}+u_{0j}}}\right)}^{1-yij}. \end{array}$$

For the parameter *σ*_*u*0_^2^, the full conditional distribution is denoted as

(6) $$\begin{array}{c} P\;\left(\frac{\sigma_{u0}^{2}}{\beta_{0},y_{ij}}\right)\propto \text{gamma }\left(\frac{N}{2}+N\left(\alpha -1\right),N\beta \right), \end{array}$$

where *N* = ∑*nj* is the total number of individual respondents interviewed in all regions of the country and *α* and *β* are scale and shape parameters, respectively, which are both fixed constants.

*(2) Variance Component Model*. The variance component model has the form

(7) $$\begin{array}{c} Y_{ij}=\beta_{0}+u_{0j}+\epsilon_{ij}, \end{array}$$

where *Y*_*ij*_ is the utilization of health care services of the  *i*^th^ women in *j*^th^ region; *u*_0*j*_ is regional level random effects that are i.i.d. normally distributed with mean zero and constant variances *σ*_*u*_^2^. *ϵ*_*ij*_ is errors that are i.i.d. normally distributed with zero means and constant variances *σ*_*e*_^2^ and  *β*_0_  is the overall mean.

The model decomposes the total variance into two the region and women levels, representing between and within region variabilities in the utilization of antenatal care services [14]. The interclass correlation (ICC) measures the correlation between observations within cluster (region) as

(8) $$\begin{array}{c} \text{ICC}=\frac{\sigma_{u}^{2}}{\sigma_{u}^{2}+\sigma_{\varepsilon }^{2}}, \end{array}$$

where *σ*_*u*_^2^ is the “between-region” variance and *σ*_*ε*_^2^ is the “between-mothers” variance.

*(i) Bayesian Multilevel Analysis of Random Intercept Models*. In the random intercept logistic regression model, the intercept is the only random effect meaning that the groups differ with respect to the average value of the response variable, but the relation between explanatory and response variables cannot differ between groups. It represents the heterogeneity between groups in the overall response.

We now assume that there are variables which are a potential explanation for observed success or failure. These variables are denoted by

(9) $$\begin{array}{c} X_{h}=\left\{X_{hij,}i=1,2,\cdots .n_{j},h=1,2,\cdots \cdots ,k,\text{and }j=1,2,\cdots ..,N,k\text{ is the number of predictors}\right\}. \end{array}$$

The logistic random intercept model expresses the log-odds, i.e., the logit of *p*_*ij*_, as a sum of a linear function of the explanatory variable and a random group-dependent deviation *u*_0*j*_.

(10) $$\begin{array}{c} \text{logit }\left(p_{ij}\right)=\beta_{0}+\overset{k}{\underset{h=1}{\sum }}\beta_{h}x_{hij}+u_{0j}, \end{array}$$

where *β*_0_+∑_*h*=1_^*k*^*β*_*h*_*x*_*hij*_ is the fixed part of the model, because the coefficients are fixed, the remaining part  *u*_0*j*_ is known as the random part of the model [15] and the success probability is

(11) $$\begin{array}{c} p_{ij}=\frac{e^{\beta_{0}+\sum_{h=1}^{k}\beta_{h}x_{hij}+u_{0j}}}{1+e^{\beta_{0}+\sum_{h=1}^{k}\beta_{h}x_{hij}+u_{0j}}}. \end{array}$$

For the prior and posterior distribution, it is also the same as in the above equation.

*(ii) Bayesian Multilevel Analysis of Random Coefficient Models*. Random coefficient models are ones where the coefficient of lower-level predictors is modeled as well. The random coefficient model explains unobserved heterogeneity in the effects of explanatory variables on the response variable.

Consider explanatory variables which are potential explanations for the observed outcomes. Denoting these variables by *X*_*h*_={*X*_*hij*,_ *i* = 1, 2, ⋯.*n*_*j*_, *h* = 1, 2, ⋯⋯, *k*, and *j* = 1, 2, ⋯.., N, }. Since some or all of these variables could be level-one variable, the success probability is not necessarily the same for all individuals in a given group. Therefore, the success probability depends on the individual as well as the group and is denoted by *p*_*ij*_.

(12) $$\begin{array}{c} \text{logit }\left(p_{ij}\right)=\log\left(\frac{p_{ij}}{1-p_{ij}}\right)=\beta_{0}+\overset{k}{\underset{h=1}{\sum }}\beta_{hj}{\text{x}}_{hij}+u_{0j}+u_{1j}x_{ij}. \end{array}$$

The first part of the above model, *β*_0_ + ∑_*h*=1_^*k*^*β*_*hj*_*x*_*hij*_, is the fixed part, (*u*_0*j*,_*u*_1*j*_) follows a bivariate normal distribution, and *u*_0*j*~_*N* (0, *σ*_*u*0_^2^), *u*_1*j*~_*N* (0, *σ*_*u*1_^2^), and cov (*u*_0*j*,_*u*_1*j*_) = *σ*_*u*01._

*(a) Prior Distribution*. Let us denote the parameters *β*_0_, *β*_1_, ⋯⋯⋯⋯.*β*_*k*_ and *Ω*_*u*_ as prior distribution as follows.

*P*(*β*_0_) ∝ 1, *P*(*β*_1_), ⋯⋯⋯⋯*P*(*β*_*k*_) ∝ 1 and *p*(*Ω*_*u*_) ~ inverse‐Wishartk (*s*_*u*_, *h*) distribution, where *Ω*_*u*_ is the variance-covariance matrices, *s*_*u*_ is an estimate for the true value of *Ω*_*u*_, and *k* is the dimension of *Ω*_*u*_ that denotes the degree of freedom, so this prior is essentially as uninformative prior. In statistics, the Wishart distribution is a generalization to multiple dimensions of the chi-squared distribution or, in the case of noninteger degrees of freedom, of the gamma distribution [16]. The Wishart distribution is the sampling distribution of the matrix of sums of squares and products of normal distributional assumption. Then, *Ω*_*u*_ is positive definite with probability density function given by

(13) $$\begin{array}{c} f\left(\Omega_{u}\right)=\frac{{\left|s\right|}^{h/2}}{{\left|\Omega \right|}^{\left(h+p+2\right)/2}2^{hk/2}\text{gamma}{\left(k\right)}^{h/2}}\exp\left\{-\frac{1}{2}tr\left(s^{-1}\Omega \right)\right\}. \end{array}$$

*(b) Posterior Distribution*. For the parameter *β*_0_, *β*_1_, *β*_2_, ⋯⋯⋯⋯., *β*_*k*_, the posterior distribution is given by

(14) $$\begin{array}{c} P\;\left(\beta_{\hat{h}}/\Omega_{u},y_{ij}\right)\propto \underset{ij}{\prod }\left({\left(p_{ij}\right)}^{yij}{\left(1-p_{ij}\right)}^{1-yij}\right), \end{array}$$

where $\hat{h}=0,1,2,\cdots ..,k$. For the parameter **Ω**_**u**_, the full conditional distribution is given by

(15) $$\begin{array}{c} f\;\left(\Omega_{u}/\beta_{\hat{h}},y_{ij}\right)\propto p\left.\left(y_{ij}/\Omega_{u},\beta_{h}\right)\right)\left(f\left(\Omega_{u}\right)\right). \end{array}$$

*(c) Estimation Techniques for MCMC*. Bayesian estimation was performed using the software MLwiN, a specialized program for performing multilevel analysis. The Markov chain Monte-Carlo (MCMC) method is a general simulation method for sampling from posterior distributions and computing posterior quantities of interest.

For Bayesian model selection, the deviance information criterion (DIC) that is a hierarchical modeling generalization of the AIC is used. The definition of deviance is a -2loglikelihood ratio of a reduced model compared to the full model. In Bayesian, the lowest expected deviance has the highest posterior probability. Assessing goodness of fit involves investigating how close the values are predicted by the model with that of observed values [17]. The comparison of observed to predicted values using the likelihood function is based on the statistic called deviance.

(16) $$\begin{array}{c} D=-2\sum \left[y_{i}\log\left(p_{i}\right)+\left(1-y_{i}\right)\log\left(1-p_{\left.i\right)}\right.\right], \end{array}$$

where *p*_*i*_ is the predicted value for observation *i*. The larger deviance, the poorer fitness of the model.

## 3. Results and Discussion

### 3.1. Descriptive Statistics on Usage of Antenatal Care Services

The results displayed in Table 1 below depict the percentages and counts of usage of antenatal care services with respect to socioeconomic and demographic variables. All computations were conducted in Statistical Package for Social Sciences (SPSS) version 20.0 and MLwiN version 2.02. Out of the 7171 women requested for ANC services, 2479 (34.6%) women were not receiving ANC services, while 4692 (65.4%) women were receiving ANC services at the time of data collection.

The descriptive measures in Table 1 illustrate the relationship between socioeconomic and demographic variables with the use or not use of antenatal care services. Based on the *P* value in the last column, all the variables have an association with the use or not use of antenatal care.

### 3.2. Bayesian Multilevel Logistic Regression Analysis Output

In the multilevel analysis, a two-level structure is used with regions as the second-level units and women as the first-level units. The expectation is that there would be differences in the usage of ANC services among regions. The nesting structure is women within regions with a total of 7171 women.

### 3.3. Comparisons of Model

As it is depicted in Table 2, the deviance-based chi-square test of the random coefficient of the Bayesian multilevel model is statistically significant (*P* value = 0.000^∗^) and the deviance-based chi-square test is 290.47. This implies that as compared to the model with a random intercept with the fixed explanatory variables and null Bayesian multilevel model, the Bayesian multilevel model with random coefficient is better fit. Therefore, the study has been focused on the Bayesian multilevel logistic regression for the random coefficient model.

From Table 3, the measure $\bar{D\;}$ indicates the average deviance from the complete set of iterations. $D\left(\bar{\theta }\right)$ indicates the deviance at the expected value of the unknown parameters, and pD is the estimated degrees of freedom consumed in the fit or it is the difference between the average deviance from the complete set of iterations and the deviance at the expected value of the unknown parameters. Also, the deviance information criterion is the sum of $\bar{D\;}$ and pD. For comparing models, when we use the DIC (deviance information criteria). The random intercept model reduced by 942.39 than the null model. Thus, the random intercept model with the fixed explanatory variables shows a highly significant improvement suggesting that it is better than the null model and the DIC diagnostics of the random coefficient model is reduced by 950.9 and 8.51 from the random intercept model with the fixed explanatory variables and the null model, respectively. Therefore, this smallest value of DIC indicates that Bayesian multilevel logistic regression for the random coefficient model is the most significant model as compared to the null and random intercept model with the fixed explanatory variables. Therefore, this study employs the Bayesian multilevel logistic regression for the random coefficient model.

### 3.4. Results of Bayesian Multilevel Analysis of the Null Model

The null model contains no explanatory variables, and it can be focused on assessing the heterogeneity of utilization of antenatal care services among regions. As it is displayed in Table 4, the multilevel empty model fits better than the single-level empty model for antenatal care services. The result from Table 4 indicates that the variance of the random factor is significant which indicates that there is regional variation in receiving antenatal care services.

From the result presented in Table 4 above, it is observed that the overall mean of receiving antenatal care services from a health professional is estimated as *β*0 = 1.215 and the regional variance *σ*_*u*0_^2^ is estimated as 2.830.

From the result presented in Table 4 above, it is observed that the overall mean of receiving antenatal care services from a health professional is estimated as *β*0 = 1.215 and the regional variance *σ*_*u*0_^2^ is estimated to be 2.830.

The variances *σ*_*ε*_^2^ and *σ*_*u*_^2^ in Table 4 estimate the variation among individual mother and among regions of the country. The individual (level-1) variance was fixed to *π*^2^/3 (3.29) for the logit model. Also, to examine how much variation in the utilization of ANC services of women was attributable to the region level factors, it is useful to see the intraregion correlation coefficient (ICC) = *σ*_*u*_^2^/(*σ*_*u*_^2^ + *σ*_*ε*_^2^) = 2.830/(2.830 + 3.29) = 0.4624, which measures the proportion of variance of receiving antenatal services that is between regions, not within regions. The intraregion correlation coefficient (ICC) in the intercept-only model is 0.4624 which is significant at a 5% level of significance. This means that 46.24% of the variation in utilization of ANC service exists between regions, whereas the remaining is 53.76% attributable to an individual level, i.e., within region differences.

### 3.5. Bayesian Multilevel Logistic Regression of the Random Coefficient Model

This model contains a random slope for the educational attainment of women, which means that it allows the effect of the coefficient of this variable to vary from region to region. This model is more appropriate than the random intercept model for the variables being used since it is intuitive to assume that the educational attainment of women varies from region to region.

As shown in Table 5, the Bayesian multilevel random coefficient model, some of the independent variables have a significant effect on the usage of ANC services in Ethiopia. These are the place of residence, beat, birth order, religious, husband occupation, wealth index, and husband education.

For the categorical variable place of residence in Table 5, the odds of using antenatal care service for women living in rural areas is 0.206 times than women living in urban areas. In other words, the use of antenatal care services for women living in rural areas was 79.4% less than that of women in urban areas. This indicates that women who were living in rural areas were less likely to receive ANC services than living in urban areas. The educational level of the husband was also found to be a significant contributing factor for receiving ANC services at a 5% level of significance. The odds of the educational level of the husband who had primary education were 1.547 indicating that the usage of antenatal care services for those women in this category is 54.75% more than the women having a husband with no education. Similarly, women having a husband with secondary education and higher-level education have better odds of using antenatal care services than women having a husband with no education. Mothers whose husband's educational levels were primary, secondary, and higher level were more likely to receive ANC services than women whose husbands were not educated.

The birth order in Table 5 is the other significant factor for receiving ANC services at a 5% level of significance. The odds of using antenatal care service for birth order category of women having children between 5 and 8 were 0.795 indicating a 20.5% decrease than women having children from 1 to 4. Similarly, women having children greater than eight has an odd ratio of 0.719 indicating a 28.1% decrease in the use of antenatal care services compared to women having 1-4 children. In other words, women having children between 5 and 8 and greater than eight were less likely to receive ANC services than women having children from one up to four.

The effect of attitudes towards wives beating on the use of antenatal care services was the other concern of this study illustrated in Table 5. From the analysis, it was found that the variable was found to be a significant determinant variable of receiving ANC services. The odds of using antenatal care services for beating wives that argues with husband were 0.864 indicating a 13.6% decrease compared to not beating wives that argues with husband.

The household wealth index displayed in Table 5 was also the other significant factor that affects the use of antenatal care services at a 5% level of significance. The odds of using ANC for women in the medium wealth index category were 1.571 which indicates that women in the medium wealth index category use antenatal care services 57.1% higher than women who are in the poor index category. Similarly, those women in the rich wealth index category use the antenatal care services 90% higher than the women in the poor wealth index category. This indicates that women who are in the medium and rich wealth index category were more likely to receive ANC service care as compared to women in the poor wealth index category.

The husband's occupation in Table 5 was found to be a significant determinant variable of receiving ANC services. The odds of using antenatal care services for women having a working husband were 1.421 showing a 42.1% increase in usage than women whose husband was not working. In other words, women whose husband was working were more likely to receive ANC services than women whose husband was not working.

The other concern of this study was to examine the effect of religion on the use of antenatal care services. As it is depicted in Table 5, women whose religion was Catholic, Protestant, Muslim, and other followers have odds ratios of 0.335, 0.493, 0.623, and 0.252, respectively. This indicates that women whose religion are Catholic, Protestant, Muslim, and others use antenatal care services 66.5%, 50.7%, 37.7%, and 74.8%, respectively, less than women with Orthodox religion. Thus, women that follow Catholic, Protestant, Muslim, and other followers were less likely to receive ANC services than women whose religion was Orthodox.

From Table 6, we can clearly observe that the educational attainment of women has significant contributions on receiving ANC services at a 5% level of significance. The odds of educational attainment of women who had primary, secondary, and higher education were 2.052, 3.408, and 10.612, respectively. This points out that the use of antenatal care services for women with primary, secondary, and higher education was 2.052, 3.408, and 10.612 times than women who had no education, respectively. This illustrates that women whose educational levels were primary secondary and higher level were more likely to attend ANC services than women who were not educated.

The estimated variance of intercept and slope of educational attainment varies significantly. This showed that the factor women educational level has brought a variation in the usage of antenatal care services across regions of the country. Some of the variances of the interaction terms between intercepts and slopes of explanatory variables are also found significant. Interpretation of significant covariance terms can be easily made in terms of the correlation coefficients displayed in Table 6, and the correlation matrix contains the estimated correlation between random intercepts and slopes which are negatively correlated. The negative sign for the correlation between intercepts and slopes implies that regions with higher intercepts tend to have on average lower slopes on the corresponding predictors.

### 3.6. Binary Logistic Regression Analysis Output

Binary logistic outcomes (dependent variables) are binary (dichotomous) and can be coded 0 (no use of ANC) and 1 (use of ANC).

The predictors can be continuous or dichotomous, as in regression analysis. The output of the binary logistic regression is depicted in Table 7.

Among the covariates in the model, residence, birth order, religion, husband occupation, wealth index, and husband education have a significant effect on the odds of using antenatal care services. But the remaining covariate respondent's opinion that a husband is justified in hitting or beating his wife and age of the respondents do not have a significant effect on the odds of using antenatal care services at a 5% level of significance.

### 3.7. Comparison of Bayesian Multilevel Random Coefficient and Binary Logistic Models

The goodness of fit of various statistical models can be examined using various statistical measures. These measures are used to select a suitable model among the possible candidate models. In Table 8, some model comparison measures have been listed. Among these, the most commonly used statistical measures used to select a model having good predictive capacity are AIC and BIC. The model having the smallest AIC (DIC) and BIC is considered to have a good fit and predictive performance that can minimize the difference between the actual and predicted observations.

On the basis of the AIC and DIC values in Table 8, the multilevel random coefficient model has a good fit or better predictive performance than the binary logistic regression model as both the DIC for it is much smaller compared to the AIC for the binary logistic model. The other comparison measures also confirmed that the multilevel random coefficient model has a good fit.

## 4. Discussion

This study employed the Bayesian multilevel logistic regression analysis. Women are nested within the various regions in Ethiopia in order to explain regional variations in the usage of antenatal services. We employed three multilevel logistic regression models for the response variable on the use or not use of antenatal care services. The Bayesian multilevel logistic regression empty model, Bayesian multilevel logistic regression random intercept, and Bayesian multilevel logistic regression for the random coefficient model were applied to explain regional differences in the usage of antenatal care services among women.

The place of residence was significantly associated with the usage of antenatal care services. This shows that women who were living in rural areas were less likely to receive ANC services than living in urban areas. This difference might be due to the fact that urban women are more accessible to health services and have information and education about ANC service care than women in the rural area. The findings from this study and previous studies in Ethiopia showed that there exists an association between ANC utilization and residence of mothers [18–20].

In this study, it was found that women in primary, secondary, and higher education were more likely to receive antenatal care services than women who are not educated. The study that was conducted in Ghana also showed a similar finding with this study [21]. Moreover, another study conducted in Assosa, Ethiopia, concluded that women's education is the determinant factor for the use of antenatal care services [22]. Thus, this study also confirmed that women who attended formal education are better in using antenatal care services than those who are not educated.

This study also concluded that the husband's educational level were a significant contributing factor for receiving ANC services. This indicates that mothers whose husband's educational levels were primary, secondary, and higher levels were more likely to receive ANC services than those not educated. The other studies conducted in Ethiopia also verified that women with partners having primary or higher education used ANC services more than those women who had partners/husbands with no education in Ethiopia [23].

The other factor of interest for this study was the household wealth index. It was found that the household wealth index was an important determinant factor on the usage of ANC service. This indicates that women who are from a household with medium and higher wealth quintile are more likely to utilize ANC service care than those who are from poor wealth households. This positive relationship between wealth index and antenatal care usage was also supported by a study conducted by various researchers [18, 24].

The birth order was the other determinant factor that influences the usage of antenatal care services. The study conducted by Kamau and Habtom [25, 26] also confirmed this truth. Moreover, research conducted by Habtom [26] showed that the mothers used more antenatal care during the second-order birth compared to the first-order birth but the use of antenatal care decreased with birth order more than two. This research also depicted that the higher the birth order, the lesser the usage of antenatal care.

The husband's occupation was a significant determinant variable on the usage of ANC services. This indicates that women whose husbands are working were more likely to receive ANC services than women whose husbands are not working. This finding is in agreement with the results of the previous study by Terfasa et al. [27] stating that women with husbands in working status were more likely to receive antenatal service care.

The study conducted by Abosse et al. concluded that the attitude towards beating wives that argues with husband was a significant determinant factor on receiving ANC services. This study also approved that women who believed that the husband is justified in hitting or beating his wife are less likely to receive antenatal care services. Thus, women who have positive attitudes towards beating wives that argues with husband use less antenatal care services that is also confirmed by Abosse et al. [28].

## 5. Conclusion

The study identified some socioeconomic and demographic factors that affect the utilization of antenatal service cares. Based on the findings, the upgrading of women and their husband's education is a very important factor on the utilization of antenatal care services. In this regard, all stakeholders particularly the government should act to raise women's and their husband's education level. Moreover, the government and other stakeholders working on health and related issues should strengthen the effort to improve the accessibility of health facilities, constructing roads and providing transportation services for pregnant women in rural areas. Finally, health extension workers should be given better training to identify and transfer high-risk women for better antenatal services in a health institution.

## Figures and Tables

**Table 1:** Statistical measures depicting the relationship between ANC usage and some socioeconomic and demographic variables.

Variables	Category	Count	%	Usage of ANC		Df	chi-sq	*P* val
				No (%)	Yes (%)			
Educational attainment of women	No education	4344	60.6	2018 (46.45)	2326 (53.55%)	3	742.1	*P* < 0.001
	Primary	1937	27.0	398 (20.55%)	1539 (79.45%)			
	Secondary	575	8.0	56 (9.74%)	519 (90.26%)			
	Higher	315	4.4	7 (2.22%)	308 (97.78%)			
Wealth index	Poor	3602	50.2	1777 (49.33%)	1825 (50.67%)	2	784.69	*P* < 0.001
	Medium	1022	14.3	321 (31.41%)	701 (68.59%)			
	Rich	2547	35.5	381 (14.96%)	2166 (85.04%)			
Place of residence	Urban	1505	21.0	117 (7.77%)	1388 (92.23%)	1	604.63	*P* < 0.001
	Rural	5666	79.0	2362 (41.69%)	3304 (58.31%)			
Women's occupation	Not working	4054	56.7	1593 (39.29%)	2461 (60.71%)	1	92.046	*P* < 0.001
	Working	3117	43.3	886 (28.42%)	2231 (71.58%)			
Husband occupation	Not working	674	9.4	342 (50.74%)	332 (49.26)	1	85.21	*P* < 0.001
	Working	5969	83.2	1963 (32.89)	4006 (67.11)			
	Missing	528	7.4					
Husband educational level	No education	3124	43.6	1519 (48.94)	1605 (51.38)	3	568.08	*P* < 0.001
	Primary	2156	30.1	591 (27.41)	1565 (72.59)			
	Secondary	744	10.4	109 (14.65)	635 (85.35)			
	Higher	619	8.6	86 (13.89)	533 (86.11)			
	Missing	528	7.4					
Age of respondents	15-24	1850	25.8	573 (30.97%)	1277 (69.03%)	2	68.92	*P* < 0.001
	25-34	3532	49.3	1144 (32.39%)	2388 (67.61%)			
	35-49	1789	24.9	762 (42.59%)	1027 (57.41%)			
Birth order	1-4	4547	63.4	1264 (27.81%)	3283 (72.20%)	2	262.53	*P* < 0.001
	5-8	2212	30.8	996 (45.03%)	1217 (55.02%)			
	>8	411	5.7	219 (53.28%)	192 (46.72%)			
Religion	Orthodox	2360	32.9	511 (21.65%)	1849 (78.35%)	4	309.97	*P* < 0.001
	Catholic	49	0.7	11 (22.45%)	28 (57.14%)			
	Protestant	1337	18.6	484 (36.20%)	853 (63.8%)			
	Muslim	3313	46.2	1387 (41.87%)	1926 (58.13%)			
	Other	112	1.6	76 (67.86%)	36 (32.14%)			
Decision making	Women	1179	16.4	382 (32.4%)	797 (67.6%)	1	66.04	*P* < 0.001
	Both	4120	58	1330 (32.18%)	2790 (67.8%)			
	Husband	1344	18.2	593 (44.82%)	751 (55.18%)			
	Missing	528	7.4					

Statistical significance at 5%.

**Table 2:** Bayesian multilevel logistic regression model comparison.

	Empty model	Random intercept	Random coefficient
Deviance (MCMC)	6889.59	6040.15	5979.97
Deviance based chi-square test	906.42	298.02	290.47
*P* value	*P* < 0.001	*P* < 0.001	*P* < 0.001

Statistical significance at 5%.

**Table 3:** Bayesian deviance information criteria.

$\bar{D}$	$D\left(\bar{\theta }\right)$	pD	DIC	Model
6889.60	6459.95	429.65	7319.25	Bayesian multilevel null model
6040.15	5703.45	336.71	6376.86	Random intercept model
5979.98	5606.13	5591.61	6368.35	Random coefficient model

**Table 4:** Bayesian multilevel logistic regression empty model.

Fixed part	Estimate	S.E	*Z* value	*P* value
Intercept	1.215	0.079	15.38	0.002
Random part	Estimate	S.E		
Var(*u*_0_) = *σ*_*u*0_^2^	2.830	0.273		
ICC	0.4624			

Statistical significance at 5%.

**Table 5:** Results of the random coefficient Bayesian multilevel logistic regression model.

Fixed effect	$\hat{\beta }$	$\text{Exp}\left(\hat{\beta }\right)$	S.E	*Z* value	*P* value
Constant	1.696	5.452	0.212	8.00	0.002
*Place of residence*					
Urban (ref)					
Rural	-1.578	0.206	0.171	9.228	*P* < 0.001
*Beat*					
No (ref)					
Yes	-0.146	0.864	0.067	2.179	0.002
*Age*					
15-24 (ref)					
25-34	0.124	1.132	0.105	1.181	0.167
35-49	-0.083	0.92	0.133	0.624	0.273
*Birth order*					
1-4 (ref)					
5-8	-0.23	0.795	0.093	2.473	0.002
>8	-0.33	0.719	0.159	2.075	0.026
*Religion*					
Orthodox (ref)					
Catholic	-1.094	0.335	0.451	2.426	0.034
Protestant	-0.707	0.493	0.147	4.809	0.009
Muslim	-0.473	0.623	0.134	3.529	0.020
Other	-1.375	0.252	0.313	4.393	*P* < 0.001
*Husband occupation*					
Not working (ref)					
Working	0.352	1.421	0.108	3.259	*P* < 0.001
*Wealth index*					
Poor (ref)					
Medium	0.452	1.571	0.099	4.566	*P* < 0.001
Rich	0.642	1.9	0.1	6.42	*P* < 0.001
*Husband education*					
No education (ref)					
Primary	0.436	1.547	0.083	5.253	*P* < 0.001
Secondary	0.645	1.906	0.154	4.188	*P* < 0.001
Higher	0.178	1.195	0.182	0.978	0.045

Statistical significance at 5%; ref = categorical reference.

**Table 6:** Results for fixed and random effects of the Bayesian multilevel random coefficient model.

Covariates	$\hat{\beta }$	$\text{Exp}\left(\hat{\beta }\right)$	S.E	*Z* value	*P* value
*Edu. of women*					
No education(ref)					
Primary	0.719	2.052	0.113	6.363	*P* < 0.001
Secondary	1.226	3.408	0.224	5.473	*P* < 0.001
Higher	2.362	10.612	0.502	4.705	*P* < 0.001
*Random part of coefficient: level-2* variance-covariance	Estimated variance		S.E		
Var((*u*_0*j*_) = *σ*_*u*0*j*_^2^	1.361		0.173		
Var(*u*_11*j*_)	0.440		0.181		
Cov(*u*_0*j*_, *u*_11*j*_)	-0.168		0.173		

Statistical significance at 5%; ref = reference category.

**Table 7:** Results of the binary logistic regression model.

Covariates	$\hat{\beta }$	S.E	Wald	Df	Sig.	Exp (B)
*Residence*			15.635	2	*P* < 0.001	
Urban (ref)						
Rural	-1.244	0.126	97.266	1	*P* < 0.001	0.288
*Beat*			2.713	2	0.258	
No (ref)						
Yes	-0.602	0.390	2.381	1	0.123	0.548
*Age*			5.285	2	0.071	
15-24 (ref)						
25-34	-0.344	0.075	20.883	1	*P* < 0.001	0.709
35-49	-0.455	0.136	11.166	1	*P* < 0.001	0.634
*Birth order*			23.048	2	*P* < 0.001	
1-4 (ref)						
5-8	0.455	0.136	11.166	1	*P* < 0.001	1.577
>8	0.111	0.123	0.812	1	0.367	1.117
*Religion*			125.421	4	*P* < 0.001	
Orthodox (ref)						
Catholic	-1.256	0.336	13.991	1	*P* < 0.001	0.285
Protestant	-0.761	0.089	73.755	1	*P* < 0.001	0.467
Muslim	-0.635	0.071	79.030	1	*P* < 0.001	0.530
Other	-1.552	0.237	42.982	1	*P* < 0.001	0.212
*Hus. Occu.*			35.762	1		
No (ref)						
Yes	0.427	0.092	21.622	1	*P* < 0.001	1.533
*Wealth index*			123.468	2	*P* < 0.001	
Poor (ref)						
Medium	0.568	0.082	48.128	1	*P* < 0.001	1.764
Rich	0.808	0.080	101.434	1	*P* < 0.001	2.244
*Hus. Edu.*			134.630	4	*P* < 0.001	
No Edu. (ref)						
Primary	1.054	0.125	71.367	1	*P* < 0.001	2.868
Secondary	0.904	0.152	35.218	1	*P* < 0.001	2.470
Higher	0.155	0.335	0.213	1	0.644	1.167
Constant	1.367	0.175	60.958	1	*P* < 0.001	3.925

Statistical significance at 5%; ref = categorical reference.

**Table 8:** Statistical measures used to compare the goodness of fit of the binary logistic and multilevel random coefficient model.

Model comparison measures	Binary logistic regression	Multilevel random coefficient model
-2∗log likelihood	-7593.75	-5673.29
Deviance based on Chi-square	1222.079	290.47
AIC (DIC)	7631.749	6368.35

## Data Availability

In this study, we used the information from the fourth Demographic and Health Survey conducted of Ethiopia from January 18, 2016, to June 27, 2016 by Central Statistical Agency (CSA) focusing on all women who are in the reproductive age group (aged from 15-49 years).

## Abbreviations

AIC: Akakie information criterion
ANC: Antenatal care
BIC: Bayesian information criterion
CSA: Central Statistical Agency
DC: Delivery care
DIC: Deviance information criterion
EDHS: Ethiopian Demographic and Health Survey
ICC: Interclass correlation
MCMC: Markov chain Monte-Carlo
PNC: Postnatal care
SPSS: Statistical Package for Social Sciences
WHO: World Health Organization.
